# The Resting-State Electroencephalogram Microstate Correlations With Empathy and Their Moderating Effect on the Relationship Between Empathy and Disgust

**DOI:** 10.3389/fnhum.2021.626507

**Published:** 2021-06-28

**Authors:** Meng Zhang, Zhaoxian Li, Li Wang, Shiyan Yang, Feng Zou, Yufeng Wang, Xin Wu, Yanyan Luo

**Affiliations:** ^1^Department of Psychiatry, Henan Mental Hospital, The Second Affiliated Hospital of Xinxiang Medical University, Xinxiang, China; ^2^Department of Psychology, Xinxiang Medical University, Xinxiang, China; ^3^School of Nursing, Xinxiang Medical University, Xinxiang, China

**Keywords:** empathy, disgust, resting-state EEG, microstates, moderating effect, aDMN, DAT

## Abstract

Humans have a natural ability to understand the emotions and feelings of others, whether one actually witnesses the situation of another, perceives it from a photograph, reads about it in a fiction book, or merely imagines it. This is the phenomenon of empathy, which requires us to mentally represent external information to experience the emotions of others. Studies have shown that individuals with high empathy have high anterior insula and adjacent frontal operculum activation when they are aware of negative emotions in others. As a negative emotion, disgust processing involves insula coupling. What are the neurophysiological characteristics for regulating the levels of empathy and disgust? To answer this question, we collected electroencephalogram microstates (EEG-ms) of 196 college students at rest and used the Disgust Scale and Interpersonal Reactivity Index. The results showed that: (1) there was a significant positive correlation between empathy and disgust sensitivity; (2) the empathy score and the intensity of transition possibility between EEG-ms C and D were significantly positively correlated; and (3) the connection strength between the transition possibility of EEG-ms C and D could adjust the relationship between the disgust sensitivity score and the empathy score. This study provides new neurophysiological characteristics for an understanding of the regulate relationship between empathy and disgust and provides a new perspective on emotion and attention.

## Introduction

Scientists believe that empathy is an important part of human communication that puts us at our best in a world of diversity. Empathy is commonly defined as the understanding of the feelings and emotional states of others (Singer, 2006). It is a complex and multilevel concept, which researchers have many different views on the processing of empathy. Zaki and Ochsner (2012) concluded that the three related processes are: experience sharing, mentalizing, and prosocial concern. Experience sharing refers to indirectly experiencing the internal state of targets (Gallese et al., 2004), mentalization refers to explicitly considering the state of targets and the source of their emotions (Leslie et al., 2005), prosocial concern refers to expressing feelings about targets (such as reducing their pain) (Danielbatson, 2011). It is not difficult to see that experience sharing is a process of getting experience from the outside to the inside, mentalization involves bringing the external stimulus into the individual, and prosocial behavior involves expressing empathy of an individual from the inside to the outside. The empathetic ability to share the feelings of others ultimately results in a better understanding of the present and future mental states and actions of the people around us, which may promote prosocial behavior. Scholars who support this theory have found that patients with medial pre-frontal lobe injury have not only significantly lower self-reported cognitive empathetic ability compared with health control but also poor performance in emotional recognition and theoretical psychological tasks (Singer et al., 2004; Shamay-Tsoory, 2009). Furthermore, empathy is highly correlated with the development of self. Researchers have different views on the relationship between empathy and self. Carlozzi et al. (1983) found that subjects with higher levels of self-development have significantly higher empathy scores than did those with lower levels. Francesca's experiment (Fiori et al., 2017), however, suggested that high empathy and heightened bodily self-awareness might increase a self-centered perspective and limit altruistic acts.

The self is indivisibly related to disgust. Miller (2013) discussed the relationship between disgust and self, arguing that disgust is a barrier to protecting the self, or a boundary between the body and the self, which determines what enters the self and what does not. Rüsch et al. (2011) found that the self can activate disgust, and through the influence of implicit cognitive schemas on perception and behavior, they found that a person with a self-concept that includes a disgust tendency is more susceptible to the influence of stimuli associated with disgust and emotional disorders. Disgust is a negative emotion that repels or suppresses impure stimuli from the outside world and protects the self from harm. Disgust originates from the sensory organs of the body as a strong revulsion caused by the ingestion of an unpleasant object (contaminant) in the mouth (Krolak-Salmon et al., 2003). Disgust can also be a negative emotional experience induced by unpleasant stimuli. As one of the basic emotions, disgust has a unique facial expression and physiological response, which are manifested as lip lift, nose contraction, frowning, vomiting, decreased heart rate, and increased skin electricity, accompanied by the phenomenon of slow breathing (Calder et al., 2000; Gaag et al., 2007).

In addition to both being self-related, disgust and empathy have some indirect neural bases. It has been found that individuals with high empathetic ability have stronger activation of the sensory system. Singer (2006) found that scores of subjects on the empathic attention dimension of the Interpersonal Reactivity Index (IRI) (Davis, 1983) could positively predict the activities of the anterior insula and dorsal anterior cingulate gyrus during pain empathy response. The anterior insula, which is involved in the processing of unpleasant taste stimuli, is also strongly associated with the processing of disgust. Since disgust originates from people refusing to take unpleasant taste stimuli into the mouth, it is not surprising that the disgust expression activates the insula (Phillips et al., 1997). Dimberg et al. (2011) found that more empathic individuals showed greater facial muscle contraction in response to angry faces compared to less empathic individuals. Jabbi et al. (2007) found that empathy scores of participants were predictive of their gustatory anterior insula and adjacent frontal operculum activation while witnessing both the pleased and disgusted facial expressions of others. However, few studies have directly explored the relationship between disgust and empathy.

Therefore, in this study, we explored the relationship between empathy and disgust and then further studied the neural functional network that regulates their relationship. Previous research on the brain functional networks mainly used the method of resting functional MRI (fMRI) (Van Den Heuvel and Pol, 2010). On a much shorter timescale than fMRI, electroencephalogram (EEG) data display sub-second (~60–120 ms) periods of stable brain states that are consistent across time and individuals (Koenig et al., 2002; Lehmann et al., 2005). These quasi-stable periods are called microstates (Lehmann et al., 1987). They are recorded by multichannel EEG and are characterized by a unique topography of electric potentials over the entire channel array. In recent years, studies on large-scale dynamic neural networks in the resting state of the brain have made abundant achievements in revealing behavioral differences between individuals and within individuals (Nishida et al., 2013; Zanesco et al., 2020). Despite the abundance of maps in the multichannel record, the researchers selected prototypes of four microstates that make up most of the global topography (Lehmann et al., 2009). The temporal characteristics of the four brain networks measured in this way refer to: duration characteristics, frequency characteristics, relative coverage to other microstates, and the transition possibilities characteristics of each microstate (Michel and Koenig, 2018). Each characteristic can be interpreted based on the underlying neural activities. For instance, the duration, which represents the temporal stability of each microstate, while the frequency of EEG occurrence may represent the tendency of microstates to be active. The transition possibilities extract the asymptotic behavior of transitions between microstates (i.e., the likelihood of switching between different microstates). This method can assess the function of large-scale brain networks by taking into account signals from the entire cerebral cortex. To date, no study has attempted to investigate the neurophysiological characteristics for regulating empathy and disgust by using the EEG microstate (EEG-ms) analysis. Based on previous studies on disgust and empathy, we believe that it is feasible to explore the relationship between empathy and disgust with microstates and the neural functional network regulating the relationship. We hypothesized that there may be a positive correlation between empathy and disgust.

## Method

### Participants and Procedure

About 196 right-handed students (116 females, mean age ± SD = 18.291 ± 0.815 years) from the Xinxiang Medical University participated in the monetary compensation after giving the informed consent approved by the Ethics Committee of the Xinxiang Medical University. Participated in the study as part of our ongoing longitudinal research project to investigate the relationship between the brain, mental health, and cognition. After completing all data collection, participants will be paid 500 yuan. The participants did not suffer from cold or cough, stay up late, had no history of neurological/psychiatric disease, or did not take any medication that could affect the experiment.

When the participants came to the laboratory, they were first filled in the Disgust Scale (DS) (Haidt et al., 1994) and IRI to assess the level of disgust sensitivity and empathy. The 32-item DS is based on self-report and is divided into two parts. One part has 16 true/false items, which requires the participants to answer according to their own situation. Three of the true-false items are reverse scored. The other part has 16 items, which will be rated on a three-point scale. The 16 items assess the extent to which participants found the experience disgusting: not disgusting at all (0), slightly disgusting (1), or very disgusting (2). Haidt et al. (1994) suggested that researchers may calculate a total score for the overall disgust sensitivity by summing the responses to the 32 items. Participants ranged in age from 18 to 52 (*M* = 19.25, *SD* = 2.55). Correlation coefficients between subscales ranged from 0.34 to 0.64. A score higher than 16 is high disgust sensitivity, otherwise low disgust sensitivity. The IRI consists of 28 items and measures four dimensions of empathy: Perspective Taking (PT), Fantasy (F), Empathic Concern (EC), and Personal Distress (PD). Each dimension is composed of seven items. All of these items are scored on a five-point summative rating scale ranging from 1 *does not describe me well* to 5 *describes me very well*. This scale is based on self-report, which requires the participants to make a 1–5 rating according to their own situation. The internal consistency reliability is 0.71–0.77, and the retest reliability is 0.62–0.80. The participants were then asked to wash their hair three times for the full EEG impedance reduction and then to sit in a separate room with moderate light and temperature for the EEG data collection after the hair was completely blow-dried. Participants were asked to close their eyes and rest for 5 min, during which time they remained calm and did not fall asleep.

### Electroencephalogram Data Acquisition

The EEG data were recorded from 64 Ag/AgCl scalp sites during the process of data acquisition according to the international 10–20 system using an elastic cap (Neuroscan Quik-cap device, Australia). During recording, all of the electrodes were referenced to Cz and re-referenced offline to linked mastoids. Channels for horizontal and vertical electrooculography were computed offline from electrodes recorded from the outer canthi of the eyes and from above and below the left eye, respectively. The sampling rate for collecting the EEG data was 500 Hz. Electrode impedances were under 5 kΩ.

### Electroencephalogram Microstate Analysis

The EEG data were preprocessed using EEGLAB (https://sccn.ucsd.edu/eeglab/index.php) in the MATLAB 2018b (http://cn.mathworks.com/). We checked all of the EEG data to make sure that there were no electrodes located on the neck or face and removed the portions that drifted larger at first. The data portions contaminated by the eye movements, electrocardiography, or any non-physiological artifacts were corrected using the independent component analysis (ICA). The current dataset was re-montaged against the average reference and segmented into 2,000 ms epochs, which amplitude ranged from −80 μV to +80 μV.

**Figure 1:**
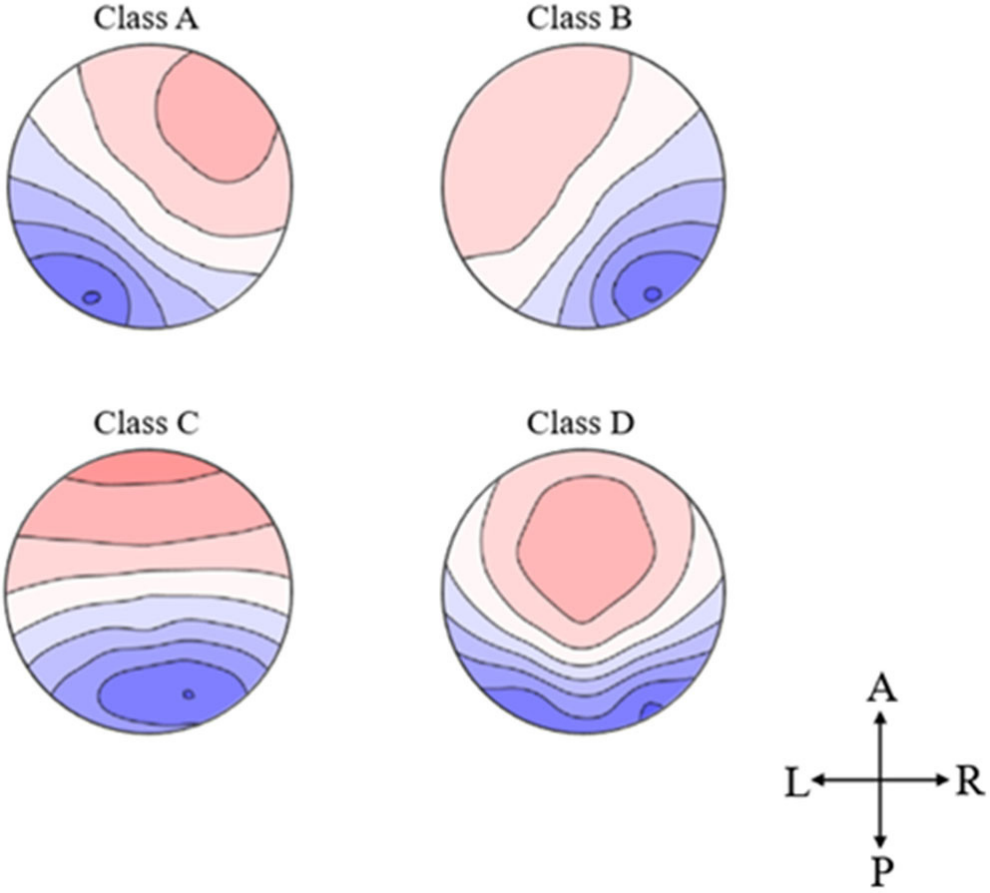
Topographical microstate maps. The figure shows the resulting four microstates, which are labeled according to previous literature. The different colors signify the different polarities. The maps are represented as seen from above (A, Anterior; P, Posterior; L, Left; R, Right).

According to previous studies, we filtered the preprocessed data by 2–20 Hz before conducting the following microstate analysis (Schlegel et al., 2012). Multichannel EEG signals were regarded as the time series of a series of instantaneous potential distribution topographic maps, which are obtained by clustering algorithm at the time points with the strongest noise ratio (Koenig et al., 2002). We first determined the global field power (GFP) and marked the peak point. The potential topographic map at the local GFP peak point had the strongest signal strength and the highest signal-to-noise ratio, which could be analyzed on behalf of the surrounding topographic map (Khanna et al., 2015). Then, the Topographic-Atomize and Agglomerate Hierarchical Clustering (T-AAHC) algorithm (Santarnecchi et al., 2017) was used to cluster the original potential topographic map obtained at the peak point of GFP. It was operated in a bottom-up manner and could be solved through a high number of randomizations. First, we used the T-AAHC algorithm to prepossess the topographies of EEG data of each participant, and it could identify clusters with similar topographical configurations. Here, the polarity of each map was disregarded, and each result cluster represented one microstate class. Second, we used the criteria implemented in CARTOOL^1^ (Brunet et al., 2011) to determine the optimal number of clusters at the individual level and group level. Later, each original map was assigned to one of the EEG-ms using the maximum spatial correlation coefficient between the tested original map and the group-level microstate maps as a criterion. Therefore, each epoch was re-expressed as an alternating sequence of microstates. During this procedure, temporal smoothing (window half-size 30 ms), a Besag factor of 10, and rejection of small time frames (when <30 ms) were applied (Murray et al., 2008). Finally, the original potential topographic maps of large numbers were grouped into four microstate topographic maps (Figure 1). We divided the collected data of 5 min (a total of 300 s) into 150 epochs, the data of each 2 s were analyzed by the microstate analysis (Gao et al., 2017; Jia and Yu, 2019), and finally the data of 2 s were obtained on average.

For the microstates of each participant, the following four parameters were computed:

1. the duration, calculated as the time coverage (in ms) during all successive original maps labeled as the same microstate class. It started and ended halfway between the last original map of the preceding microstate and the first original map of the following microstate, respectively. We applied a formula to the duration of each microstate class of each participant (Schlegel et al., 2012):
  $$\begin{array}{l} Duration_{corrected}\left(ms\right)\\ =\frac{Duration_{original}\left(ms\right)\times \left(Occurrence\;+\;Coverage\right)}{Occurrence}. \end{array}$$
2. The occurrence rate is defined as the number of occurrences of a given microstate class per second.
3. The coverage is computed as the percentage of occupied total analysis time for a given microstate class.
4. Transition possibility reflects the frequency intensity of a one-way or two-way switch between any microstate classes.

### Behavioral Data Statistics

We used the Pearson correlation coefficient to analyze the relationship between the DS and IRI in IBM SPSS v22 (IBM SPSS, Armonk, NY, USA).

### Microstate Data Statistics

In the microstate data analysis, we used the Pearson correlation to analyze the correlation coefficients between disgust and empathy scores and the four indicators of EEG-ms and the connection strength between them in each individual. The Bonferroni correction was then used to perform multiple comparisons of the *p*-values obtained. The entire data analysis was performed using the IBM SPSS v22 (IBM SPSS, Armonk, NY, USA).

A series of moderation analyses using the PROCESS macro for IBM SPSS v22 (IBM SPSS, Armonk, NY, USA; Hayes, 2013) was selected to examine the four microstates as moderators of the relation between disgust sensitivity and empathy. Custo et al. (2017) proposed that EEG-ms were the “electrophysiological correlates of a process of global, ‘conscious' integration at the brain scale level.” In this integration process, the topic of how transitions between states occur in the brain was intense discussion. The transition was a continuous transition mode, not a transient activation or inactivation of two networks, and it reflected the boundary between stability and instability in the brain networks. In the moderation model, two moderation models were built, including the disgust sensitivity score as *X* (independent variable), the empathy score as *Y* (dependent variable), and the transition strength of the microstate classes C and D as the moderating variable *M*. Finally, a bootstrapping procedure (5,000 bootstrap samples) was conducted to estimate the indirect effect of *X* on *Y* through *M*. A bias-corrected 95% CI of the indirect path that does not include zero indicates a significant indirect effect (Hayes, 2009). The Johnson-Neyman technique was selected in the PROCESS macro to calculate the critical value of the continuous quantitative adjustment variable when the simple slope was significant (Wu et al., 2020).

## Result

### Behavioral Data Results

**Figure 2:**
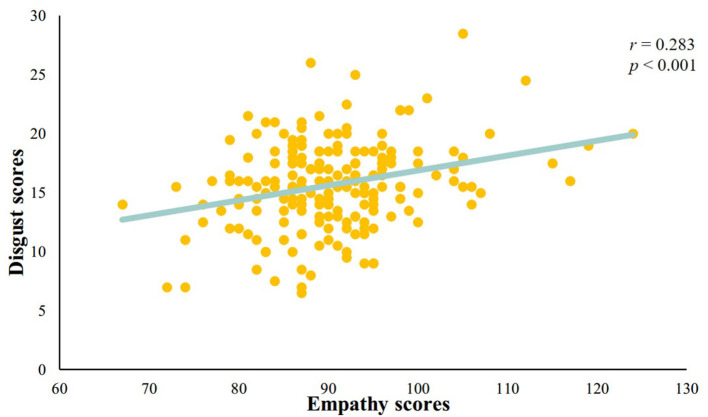
A significant positive correlation between disgust sensitivity score and empathy score.

We analyzed the correlation between the disgust sensitivity score and the empathy score. The results showed a significant positive correlation between the disgust sensitivity score and empathy score (*r* = 0.283, *p* < 0.001; see Figure 2).

### Microstate Results

**Figure 3:**
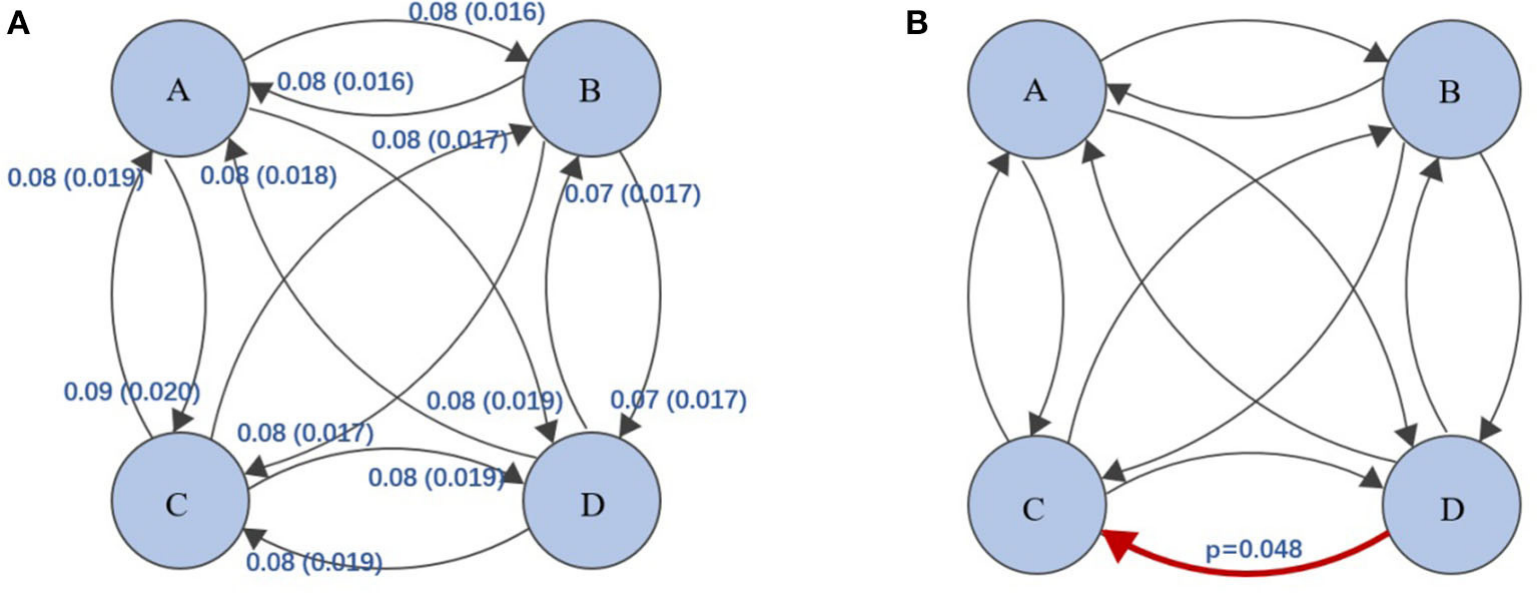
**(A)** Transition probabilities and standard deviations between the four classes of EEG-ms, e.g., class A → class B, transition probability = 0.08, standard deviation =0.016. **(B)** The red arrow represent the transition probability of class D to class C, and it was significantly positively correlated with the empathy score (*p*-values corrected for multiple comparisons using Bonferroni). The level of significance was set to *p* < 0.05.

First, we plot the topographic map of the participants under four microstates. Then, we analyzed the correlations among the disgust sensitivity score, empathy score, and the four states of current duration (CD), occurrence, contribution, and transition possibility. We found that the connection between microstates class C and class D under transition possibility was positively correlated with the empathy score. The transition possibilities of class C and class D were significantly positively correlated with the empathy score [class C to class D: Tr (C → D), *r* = 0.178, *p* = 0.013; D to C: Tr (D → C), *r* = 0.209, *p* = 0.003]. After correcting for multiple comparisons using the Bonferroni correction, the adjusted *p*-values were 0.104 and 0.048 for Tr (C → D) and Tr (D → C), respectively (see Figure 3). The descriptive statistical measure of the average duration, occurrence, and contribution of EEG-ms was shown in Table 1.

**Table 1:** Descriptive statistical of the average duration, occurrence and contribution of EEG-ms.

	**Class A**		**Class B**		**Class C**		**Class D**	
	***Mean***	***SD***	***Mean***	***SD***	***Mean***	***SD***	***Mean***	***SD***
Duration	0.074	0.011	0.071	0.010	0.073	0.011	0.071	0.012
Occurrence	3.78	0.593	3.63	0.615	3.85	0.521	3.70	0.618
Contribution	0.26	0.049	0.24	0.044	0.26	0.042	0.24	0.048

**Figure 4:**
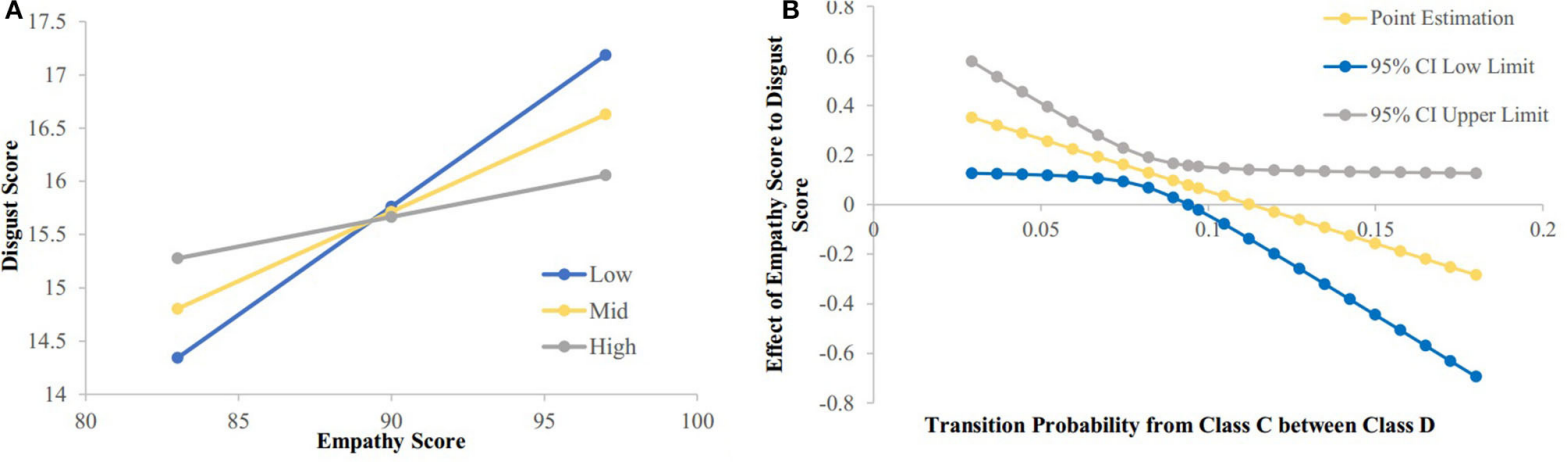
The relationship between empathy score and disgust score were modulated by possibility of transition from class C to class D. **(A)** Simple slope analysis results showed that: when the disgust score was low (mean-1sd) (sd, standard deviation), the empathy score was significantly positively correlated with the possibility of transition from class C to class D (*p* < 0.001); when the disgust score was high (mean + 1sd), the empathy score was no significant correlated with the possibility of transition from class C to class D. **(B)** Neyman results showed that the disgust score was significantly positively correlated with the empathy score when possibility of transition from class C to class D was below 0.094.

The regression analysis was used to analyze the moderating effect of the transition strength between microstate class C to class D and class D to class C on the relationship between the empathy score and the disgust sensitivity score. The results showed that the transition from class C to class D had a significant moderating effect on the relationship between the empathy score and disgust sensitivity score [*F*_(1, 192)_ = 4.0493, *p* = 0.0456, Δ*R*^2^ = 0.0189]. Meanwhile, the transition from microstate class D to class C had no significant effect on the relationship between the empathy score and disgust sensitivity score [*F*_(1, 192)_ = 3.3108, *p* = 0.0704, Δ*R*^2^ = 0.0155]. The simple slope analysis results showed that when the disgust score was lower (mean−1SD), the empathy score was significantly positively correlated with the possibility of transition from class C to class D [β = 0.203, *t* = 4.264, *p* < 0.001]; when the disgust score was higher (mean + 1SD), the empathy score was no significant correlated with the possibility of transition from class C to class D [β = −0.0556, *t* = 1.147, *p* = 0.2526]; The Johnson-Neyman results showed that the disgust score was significantly positively correlated with the empathy score when the possibility of transition from class C to class D was below 0.094 (76.02% of our sample; see Figure 4).

## Discussion

In this study, we used the microstate analysis of EEG to investigate the neural processing of disgust sensitivity and empathy. First, we found a significant positive correlation between the disgust sensitivity score and the empathy score. Second, there was a significant positive correlation between the empathy score and the connection of microstate D to C. Finally, the moderating effect analysis showed that the connection strength between microstate C and microstate D could adjust the relationship between the disgust sensitivity score and the empathy score.

The study found a significant positive correlation between the disgust sensitivity score and empathy score; in other words, individuals with higher empathy have higher levels of disgust sensitivity. Tullett et al. (2012) found that the verbal and facial expressions of disgust toward graphic images of suffering predicted levels of EC in those same individuals. Rymarczyk et al. (2019) presented 46 subjects with facial expressions of disgust, happiness, and fear and recorded the facial muscle responses, brain activity, and empathy, finding high empathy for disgust, especially in individuals with high empathy. Empathy is thought to be supported by two distinct but interacting processes (Kupfer, 2018). The mirror neurons first enable the observer to understand the internal state of others through indirect experience and then put himself in the behavior and situation through observation and imagination in the perspective of others (Keysers and Gazzola, 2006; Waytz and Mitchell, 2011; Zaki and Ochsner, 2012; Paulus et al., 2013). As a defensive emotion, disgust has a characteristic withdrawal and rejection response and functional expressive changes in which nostrils narrow (a defense against penetration), the mouth closes (to prevent incorporation or promote ejecting contaminants), salivation is increased (to dilute pollutants), and the throat constricts (to prevent swallowing) (Valerie et al., 2011; Tybur et al., 2013). By this token, empathy is a process in which the individual internalizes an external emotion and expresses their internal emotional state outward, whereas disgust is a process in which the individual inhibits an unpleasant external stimulus. Both are associated with external stimuli, and individuals with high empathy are sufficiently sensitive to both internal and external stimuli of the self and having high sensitivity to disgust. This explains our results, which show a positive correlation between empathy and disgust sensitivity. Our results extend the existing research and further refine the direct relationship between empathy and disgust.

In the resting EEG results, there was a positive correlation between the empathy score and the transition possibilities of class D to class C. The functional connections of the brain in the resting state, the consistent spontaneous fluctuations of the blood-oxygen-level-dependent (BOLD) response, have been identified in a wide range of functional cortical networks (resting-state networks) (Britz et al., 2010). Britz et al. (2010) showed that microstates can be considered the electrophysiological correlate of resting-state networks (RSNs) identified with fMRI, which suggested that the momentary scalp configuration represents the activity in a specific neurocognitive network. They pointed out that microstates A, B, C, and D correspond to RSNs related to the speech processing (Mantini et al., 2007), the visual network (Lehmann et al., 1998; Britz et al., 2010), the anterior default mode network (aDMN), which is a portion of the DMN (Taylor et al., 2009), and the dorsal attention network (DAT), respectively. Class C has been shown to be correlated with the brain regions responsible for the self-referential mental activity. An increase in the self-referential processes in DMN has been shown to be closely related to depression (Cédric et al., 2009; Sheline et al., 2009). The research of Zanesco et al. (2020) showed that slower reaction times but better response conflict resolution were associated with more frequent occurrences of class D. And these were in line with studies linking microstate D with the activity of a frontoparietal attentional control network. In our results, it can be said that the higher the empathy score, the stronger the connection from the DAT to aDMN. Furthermore, the aDMN and DAT were two basic features of consciousness. The aDMN was an internally directed system that correlates with the consciousness of self, and the DAT is an externally directed system that correlates with the consciousness of the environment. Although the results of the correlation analysis between empathy scores and Tr (C → D) were not corrected by multiple comparisons, we believe that there might be a correlation trend between them. There was no doubt that empathy, as an emotion that drives one to identify the mental status of other individuals, has a strong relationship with the aDMN which represent the body by combining interoceptive information with emotional information. When attention was transferred to the external stimuli, the aDMN related to the internal consciousness is suppressed, while the DAT, which moderates the top-down-guided voluntary allocation of attention, is activated. The relationship between the aDMN and DAT can be called anticorrelated. Studies have shown that under the general anesthesia (Bonhomme et al., 2016) and in neuropathology patients with disturbance of consciousness (Threlkeld et al., 2018), the aDMN is anticorrelated with the DAT activity and diminished. Furthermore, experience sharing and mentalizing, the processing of empathy summarized by predecessors, showed that empathy is the process of internalizing the external situations and experiencing the outside world from the individual itself, which helps explain our results.

According to our results, the unidirectional connection intensity from class C to D can adjust the relationship between the individual empathy and disgust sensitivity. The emotional regulation strategy of attention allocation was highly correlated with the neural activity in the pre-frontal cortex system (Ohira et al., 2006; Hermann et al., 2014). The relationship between empathy and disgust sensitivity increases as the emotion-to-attention connection weakens. The unidirectional enhancement of the connection between aDMN and DAT is equivalent to the enhancement of the transition from the internal state to an external stimulus. This process may enhance the mentalization and prosocial behavior of empathy, but it may not moderate the disgust experience caused mainly by external stimuli. Therefore, the one-way connection between aDMN and DAT is strengthened, then weakening the relationship between empathy and disgust. Our results revealed for the first time the relationship of aDMN and DAT with empathy and disgust, which has important implications for the study of the neurophysiological mechanisms of empathy and disgust processing. In addition, studies have shown that individuals with high disgust sensitivity are more likely to be affected by disgust stimuli and emotion-related disorders. Combined with our results, we presume that individuals with high empathy ability may be more likely to be affected by emotion-related disorders. This provides a new way to explore the relationship between empathy- and emotional-related disorders.

## Conclusion

This experiment explored the neural processing of disgust sensitivity and empathy by using the microstate analysis of EEG. It was concluded that disgust sensitivity is highly positively correlated with empathy, the empathy is significantly positively correlated with the connection from DAT to aDMN, and the connection strength between aDMN and DAT could moderate the relationship between the disgust sensitivity and the empathy.

This study was the first to use the EEG-ms to explore the relationship between disgust and empathy of the interoceptive emotion, which opens up a new way to explore individual differences in disgust sensitivity. Although there were some important and robust evidence for us to understand the relationship between disgust sensitivity and empathy, the limitation should be considered. The EEG-ms, as a method to reflect the microstate of the brain, is simple, feasible, and efficient, but the spatial construction ability is not as intuitive as MRI. And MRI can be used in the future to further verify the relationship between disgust and empathy. In addition, subjects were recruited for the diagnosis of mental illness through self-report, and the Structured Clinical Interview for DSM were not conducted. Although they are healthy college students, the possibility of mental diseases, such as anxiety and depression, cannot be completely excluded. In the follow-up study, a professional clinical interview with doctors may be required to screen the subjects and carry out strict control of mental diseases.

## Data Availability Statement

The raw data supporting the conclusions of this article will be made available by the authors, without undue reservation.

## Ethics Statement

The studies involving human participants were reviewed and approved by the Xinxiang Medical University Ethics Committee. The patients/participants provided their written informed consent to participate in this study.

## Author Contributions

MZ involved in conceptualization, methodology, formal analysis, investigation, data curation, writing—review and editing, and visualization. ZL contributed to methodology, formal analysis, investigation, data curation, writing—original draft, review and editing, and visualization. LW, SY, FZ, and YW involved in investigation and writing—review and editing. XW contributed to methodology, formal analysis, investigation, data curation, writing—review and editing, and visualization. YL involved in the investigation, writing—review and editing, and supervision. All authors contributed to the article and approved the submitted version.

## Conflict of Interest

The authors declare that the research was conducted in the absence of any commercial or financial relationships that could be construed as a potential conflict of interest.

## Footnotes

^1^The Cartool (https://sites.google.com/site/cartoolcommunity/) is a free software which was developed by the Functional Brain Mapping Laboratory in University of Geneva and could be used to perform EEG/ERP microstate analysis.
